# Bioinformatic Analysis Reveals the Association of Human N-Terminal Acetyltransferase Complexes with Distinct Transcriptional and Post-Transcriptional Processes

**DOI:** 10.1007/s10528-024-10860-z

**Published:** 2024-06-12

**Authors:** C. Koufaris, C. Demetriadou, V. Nicolaidou, A. Kirmizis

**Affiliations:** 1 Epigenetics and Gene Regulation Laboratory, Department of Biological Sciences, University of Cyprus, 2109 Nicosia, Cyprus; 2 Department of Life Sciences, University of Nicosia, Nicosia, Cyprus

**Keywords:** N-terminal acetyltransferases, Co-expression, Protein complex, Post-transcriptional, Regulation, Pathways

## Abstract

N-terminal acetyltransferases (NAT) are the protein complexes that deposit the abundant N-terminal acetylation (Nt-Ac) on eukaryotic proteins, with seven human complexes currently identified. Despite the increasing recognition of their biological and clinical importance, NAT regulation remains elusive. In this study, we performed a bioinformatic investigation to identify transcriptional and post-transcriptional processes that could be involved in the regulation of human NAT complexes. First, co-expression analysis of independent transcriptomic datasets revealed divergent pathway associations for human NAT, which are potentially connected to their distinct cellular functions. One interesting connection uncovered was the coordinated regulation of the NatA and proteasomal genes in cancer and immune cells, confirmed by analysis of multiple datasets and in isolated primary T cells. Another distinctive association was of NAA40 (NatD) with DNA replication, in cancer and non-cancer settings. The link between NAA40 transcription and DNA replication is potentially mediated through E2F1, which we have experimentally shown to bind the promoter of this NAT. Second, the coupled examination of transcriptomic and proteomic datasets revealed a much greater intra-complex concordance of NAT subunits at the protein compared to the transcript level, indicating the predominance of post-transcriptional processes for achieving their coordination. In agreement with this concept, we also found that the effects of somatic copy number alterations affecting NAT genes are attenuated post-transcriptionally. In conclusion, this study provides novel insights into the regulation of human NAT complexes.

## Introduction

**Fig.1:**
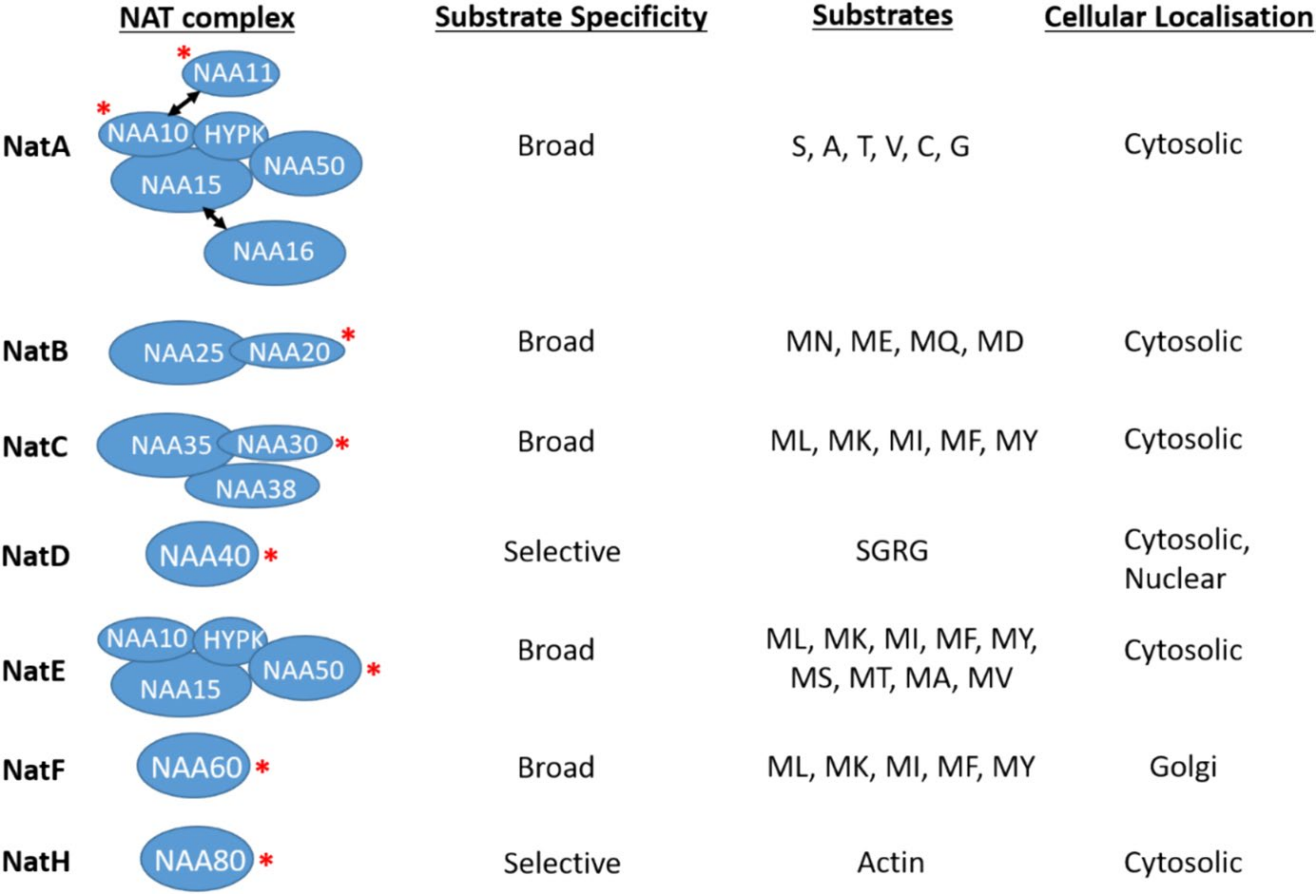
Overview of the composition, substrate repertoire and specificity, and cellular localisation of human NAT complexes, adapted from (Aksnes et al. 2019). The red star indicates the catalytic enzyme in each complex. In the case of the NatA complex, the paralogs NAA11 and NAA16 have also been shown to be able to be incorporated in place of NAA10 and NAA15, respectively. “Substrate specificity” refers to whether the NAT complex acts on one/few proteins (Selective) or to much larger repertoires (Broad). “Substrates” refer to the N-terminal amino acid residue(s) that are acted upon by the given Nat complex. Although NatA and NatE are termed cytosolic, it should be noted that their catalytic enzymes NAA10 and NAA50 have also been reported as localised in the nucleus as well. NAA11 and NAA16 are NAT paralogs that have been shown to be able to substitute for NAA10 and NAA15 in the NatA complex

N-terminal acetylation (Nt-Ac), the addition of an acetyl group to the N-terminal α-amino group of proteins, has been estimated to occur on approximately 60% of yeast and 80% of mammalian proteins (Arnesen et al. 2009; Ree et al. 2018). The Nt-Ac modification is uniquely deposited by the evolutionarily conserved family of N-terminal Acetyltransferases (NAT). Each NAT complex is composed of at a minimum a catalytic enzyme, while some are also known to contain auxiliary subunits. There are seven known human NAT complexes (NatA, NatB, NatC, NatD, NatE, NatF, and NatH) which differ in their composition, substrate repertoire, and cellular localisations. The NatA-C and NatE complexes contain auxiliary subunits that can act as ribosome anchors and/or modulate substrate specificity of the complexes. For NatA, its auxiliary subunits are NAA15, NAA50, and HYPK; for NatB, it is NAA25; and for NatC, it is NAA35 and NAA38. The human genome also encodes paralogs of NAA10 and NAA15, termed NAA11 and NAA16, respectively, that can also be incorporated into the NatA complex (Aksnes et al. 2019). (Fig. 1). A plausible explanation for the observed variety of eukaryotic NAT complexes is that this allows them to more effectively execute their distinct and diverse cellular functions.

Although the molecular significance of Nt-Ac was initially unclear, it is now known that Nt-Ac can affect protein stability and turnover, folding and aggregation, protein–protein interactions, or subcellular localisation (Aksnes et al. 2023, 2019; Ree et al. 2018). At the level of cells and organisms, Nt-Ac has been reported to affect diverse biological processes including autophagy (Shen et al., 2021), beige adipocyte-mediated thermogenesis (Lee et al., 2019), viral replication (Oishi et al., 2018), genetic diseases (McTiernan et al. 2022, 2020; Morrison et al. 2021; Muffels et al. 2021; Ree et al. 2019), cellular ageing (Molina-Serrano et al., 2016), and carcinogenesis (Demetriadou et al., 2019; Jung et al., 2020; Koufaris and Kirmizis 2020; Mughal et al., 2015). Notably, no N-terminal deacetylate has been identified so far, suggesting that this modification may be irreversible once deposited on the N-terminus of proteins.

Given the permanence, prevalence, and biological significance of Nt-Ac, understanding the regulation of NAT activity within cells is of primary importance. The majority of effort into the regulation of NAT complex activity so far has been invested—with notable progress achieved— into the identification of their protein subunits, how their interactions promote the distinct functions of the complexes, and the identification of protein agonists/antagonists (Deng and Marmorstein, 2021). For example, recent studies have revealed that binding of the auxiliary HYPK subunit onto NatA impacts complex activity (Gottlieb and Marmorstein 2018; Miklánková et al. 2022; Weyer et al. 2017), whilst the catalytic efficiency of NAA80 against actin increases when it is associated with the profilin proteins (Rebowski et al., 2020). Nevertheless, much less attention has been applied so far into examining the direct modulation of the abundance of NAT catalytic and auxiliary subunits through transcriptional and post-transcriptional processes. In this study, we were able to generate novel insights into the regulation of human NAT through an integrated examination of multiple transcriptomic and proteomic datasets.

## Results

### Individual NAT Display Comparable Transcript and Protein Abundance Across Human Tissue Types

As a starting point for our investigation, we compared the abundance of individual NAT across human tissue types. We reasoned that considerable differences between tissues in individual NAT abundance would potentially indicate tissue-specific regulation and function of these complexes. Conversely, a stable abundance across tissues would be supportive of predominantly tissue-independent functions. Three large publicly available transcriptomic datasets of non-pathogenic human tissues are the Genotype-Tissue Expression (GTEx) with data across 53 tissue types; the Human Protein Atlas (HPA) with data across 256 tissues; and the Functional ANnoTation of the Mammalian genome (FANTOM) with data across 60 tissues. A first consistent observation across the three datasets was the noticeable detection of all individual NAT transcripts across examined tissues (with a detection threshold of transcript per million (TPM) > 1), with the exception of *NAA11*. Regarding the latter, this finding agrees with previous reports that this is a testis-specific enzyme (Pang et al. 2011). We also showed here that transcript levels for *NAA11* are also detected in placenta samples that are represented in the FANTOM and HPA studies but not in GTEx, thus revealing the expression of this paralog in a second tissue type. Therefore, with the exception of *NAA11*, NAT transcripts are found ubiquitously across human tissues, consistent with their having essential and non-redundant functions.

**Fig.2:**
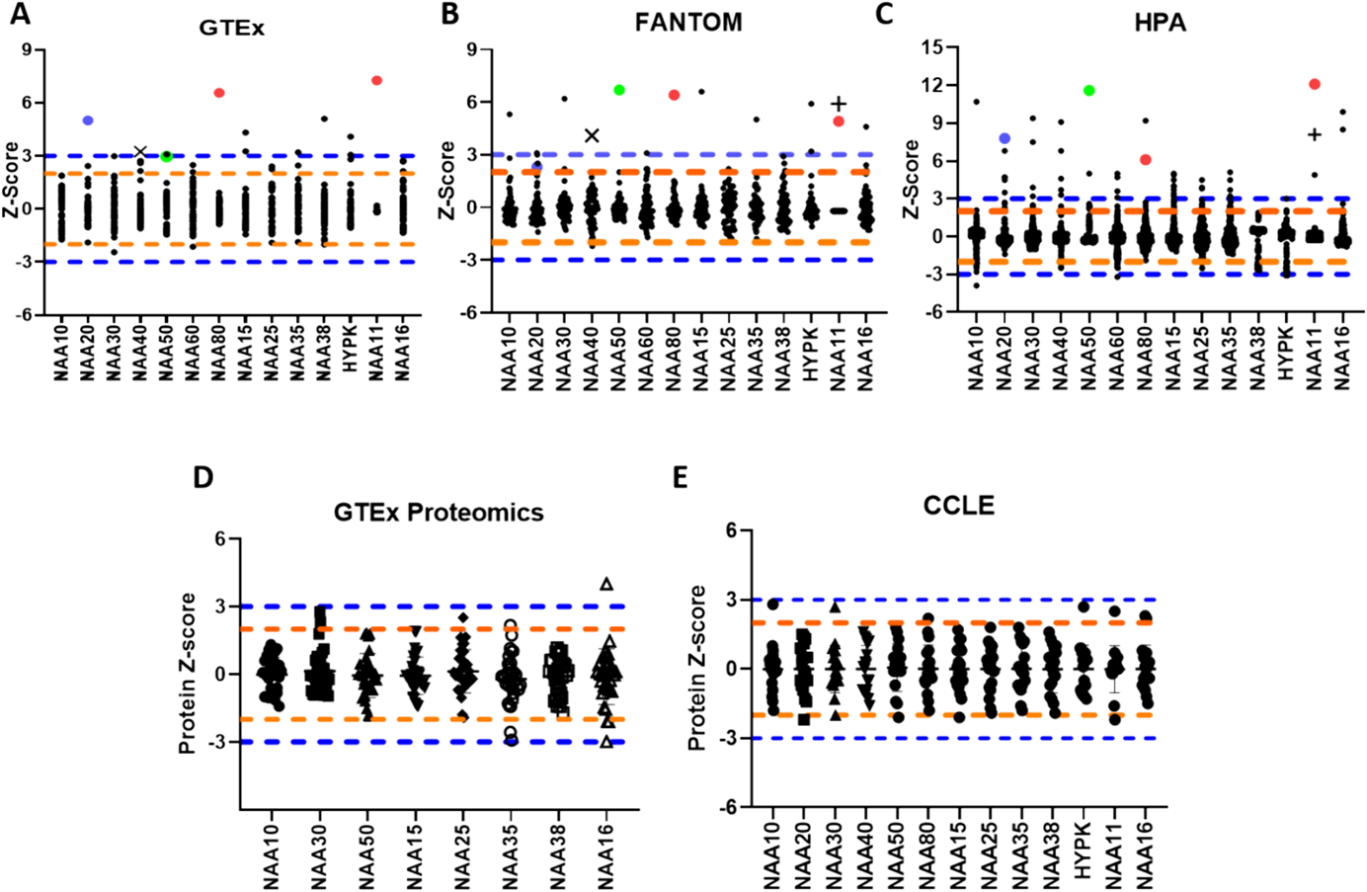
Comparable abundance of individual human NAT across cells and tissues. (**A**)–(**C**) Graphs depicting Z-scores for NAT transcripts in GTEx, FANTOM, and HPA projects calculated as described in Methods. Each plotted symbol represents the median Z-score in a given sample. Testis samples for *NAA11* and *NAA80* are depicted in red colour, oesophagus for *NAA20* in blue colour, skeletal muscle for *NAA50* in green, placenta for *NAA11* as a “ + ” symbol, and pituitary gland for *NAA40* as an “X” symbol. Placental samples were absent from GTEx and pituitary from HPA study; Protein Z-scores for detected NAT genes in (**D**) 32 GTEx tissue types or (**E**) CCLE. In the GTEx proteomics study, NAA11, NAA60 and HYPK were not reproducibly detected across tissues, while in the CCLE NAA60 was not detected. In CCLE, cell lines were grouped according to tissue of origin, when a given tissue was represented by at least five cell lines. The blue lines indicate the thresholds of ± 3 Z-scores in all graphs, and orange lines the ± 2 Z-scores

Next, we calculated the Z-scores, i.e. number of standard deviations from the mean, for the median transcript levels of each NAT across tissue types, in order to quantify variability in their abundance across tissues. A consistent finding across GTEx, HPA, and FANTOM was that NAT transcript levels were for the most part expressed at comparable levels across normal tissues, with few tissue-enriched or depleted NAT when using either ± 2 or 3 Z-score as thresholds (Fig. 2A–C). Using the more stringent ± 3 Z-score as a cut-off, the expression of *NAA11* and *NAA80* in the testis was identified as outliers in both studies. As mentioned previously, for *NAA11* this was expected as it is a testis-specific gene. Unlike *NAA11*, the *NAA80* was present in all tissues, consistent with this NAT having important role in the maturation of mammalian actin (Drazic et al. 2018), but was prominently testis-enriched (Z-scores 6.1–6.6 across the three projects). Other examples of highly tissue-enriched NATs were the expression of *NAA20* in the oesophagus and of *NAA50* in skeletal muscle. In these two cases, the Z-scores were above the threshold in 2/3 studies and borderline in the third. Moreover, the transcript levels of *NAA40* were also above 3 Z-scores in the pituitary gland within the GTEx and FANTOM studies, but this tissue was absent from the HPA study. Thus, at the transcript level, individual NAT are comparable across diverse tissues, with a few cases of tissue-enriched transcripts as noted above.

Recently, proteomic investigation of GTEx tissues has revealed that tissue-specific enrichments and depletions of proteins can also emerge post-transcriptionally (Jiang et al. 2020). We therefore next examined a quantitative proteomic dataset from 32 tissue types of GTEx. As an independent dataset, we examined the Cancer Cell Line Encyclopaedia (CCLE) proteomic dataset from 375 cell lines originating from diverse human tissues. Compared to the analogous analysis of transcriptomic datasets, proteomic analysis was restricted due to not all the NAT being detected and quantified. Specifically, NAA60 was absent from both datasets, and NAA11, NAA20, and NAA40 from the GTEx. Nevertheless, for the detected NAT, we again found comparable levels across tissues, with only one case of a tissue-enrichment or depletion, namely NAA16 in the pancreas in the GTEx study (Fig. 2D, E).

Consequently, transcriptomic and—where available—proteomic data concur that NAT display limited variability between normal tissues. This observed consistency is supportive of NAT complexes performing biologically essential and largely tissue-independent functions.

### Transcript Co-Expression Profiles Reveal the Association of NAT Complexes with Distinct Cellular pathways

The general constancy of NAT abundance across non-pathogenic human tissues does not exclude the possibility that these complexes are subjected to dynamic regulation by cellular signalling pathways, acting either across cell and tissues types or in more specialised contexts. In order to address this possibility, we performed pathway enrichment of co-expressed transcripts for each human NAT, an established and powerful methodology for investigating the regulation of genes of interest. Moreover, this approach can also allow the identification of new gene functions, based on the guilt-by-association principle (Kolberg et al., 2020; Stuart et al., 2003; Zogopoulos et al., 2022). It should be noted that since NatA and NatE complexes are composed of identical subunits, the deconvolution of their regulation is not possible by examination of expression and co-expression profiles. Consequently these are examined together as NatA/E.

**Fig.3:**
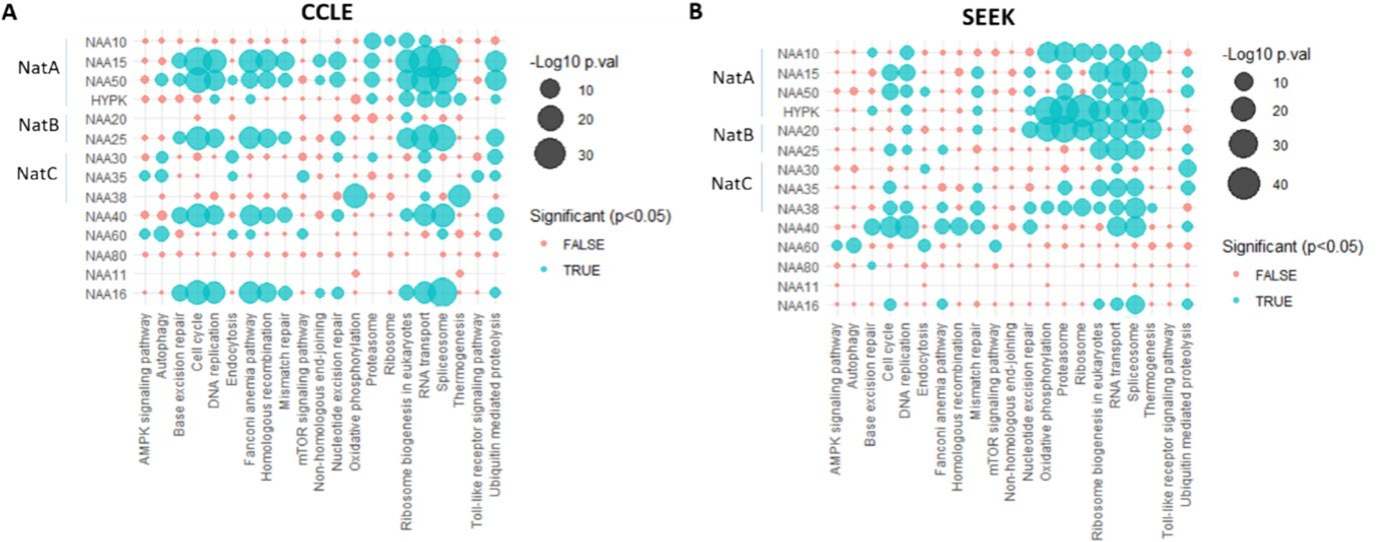
Plot depicting enrichment for selected KEGG pathways (x-axis) among significantly co-expressed transcripts for human NAT (y-axis) in (**A**) CCLE or (**B**) SEEK datasets for human NAT (y-axis). Significantly co-expressed transcripts were defined as those with Spearman’s correlations greater than 0.3 and adjusted p.val of less than 0.5 for the CCLE dataset, and the top 1000 ranked genes in the SEEK dataset. The size of the bubble corresponds to strength of the association (–log10 of the adjusted p.val,) and the colour of the bubble discriminates between significant (blue, adjusted p.val < 0.05) and non-significant (red) associations

To increase the probability of identifying the pathways associated with enriched expression of each NAT, we repeated our analysis in two independent datasets. First, the CCLE project that has generated transcriptomic data across more than 1000 human cancer cell lines. For each NAT, lists of significantly correlated transcripts—those with Spearman’s r > 0.3 and adj.p.val < 0.05—were first generated. The EnrichR tool (Xie et al., 2021) was then used to calculate the degree of overlap of these correlated gene lists with the Kyoto Encyclopaedia of Genes and Genomes (KEGG) collection of pathways. As an independent approach, we used the computational Search-Based Exploration of Expression Compendium (SEEK) search-engine to generate a list of ranked genes for each NAT according to their co-expression across thousands of microarray and RNAseq datasets. KEGG Pathway enrichment for these gene lists was then calculated again using Enrichr. Despite the difference between the CCLE and SEEK (e.g. only cell lines vs cell lines and tissues; only cancer cells vs both cancer and non-cancer cells), similar patterns of enriched pathways were identified, supporting the validity of our findings and analysis. Notably, pathway enrichment for co-expressed transcripts revealed marked differences between human NAT (Fig. 3). The major broad spectrum cytosolic NatA/B/C/E complexes were significantly associated with one or more pathways involved in protein homeostasis (“Proteasome”, “Ubiquitin mediated proteolysis”, “Ribosome”, “Ribosome biogenesis”). For the Golgi-localised broad spectrum NAA60, co-expression analysis revealed quite distinct association compared to the cytosolic complexes, with the enrichment of pathways relating to vesicle production and energy sensing (“Endocytosis”, “Autophagy”, “mTOR signalling”). Of the two highly specialised NATs, NAA40 and NAA80, the former was associated with transcripts involved in DNA replication and repair (“DNA replication”, “Cell cycle”, “Nucleotide excision repair”, “Base excision repair”, “Homologous recombination”) and RNA processing (“Spliceosome”, “RNA transport”), while this analysis revealed no commonly significant enriched pathway for the latter across the two datasets. To summarise, pathway enrichment for co-expressed transcripts and protein reveals clear differences among human NAT, indicating differences in their regulation, which could potentially relate to their distinct biological functions.

### Transcriptional Co-Regulation of NatA and Proteasomal Genes Occurs in Cancers and in Activated T Cells

**Fig.4:**
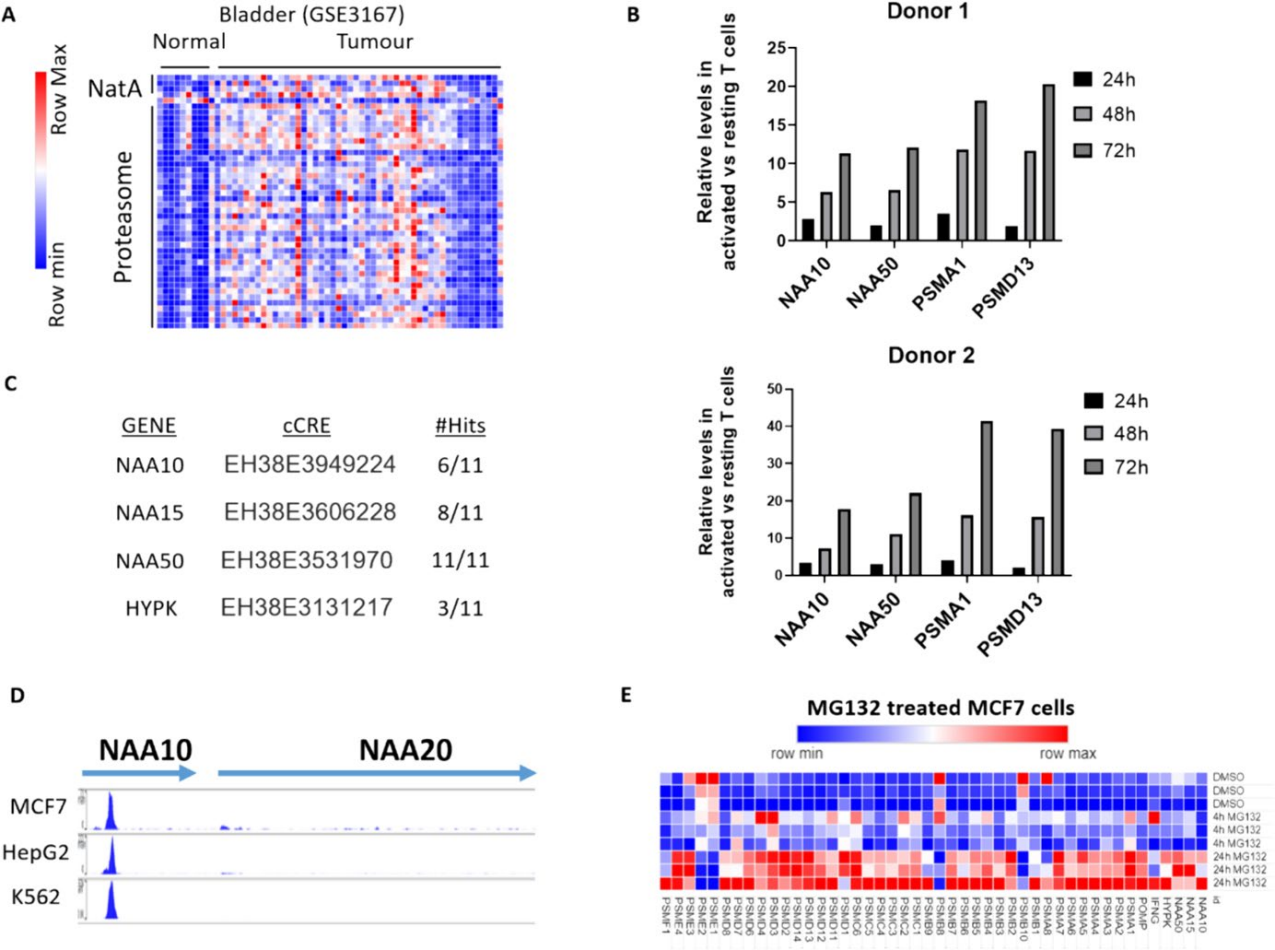
Coordinated transcription of NatA/E and proteasomal complex genes in cancer and non-cancer cells. (**A**) Example dataset from cancer study where high degree of coordination of NatA and proteasomal transcripts is shown. In the heatmap, the blue colour indicates low levels of the gene and the red colour higher expression; (**B**) Timecourse showing relative levels of *NAA10*/*NAA50* and *PSMA1*/*PSMD13* in resting vs activated T cells as assessed by qRT-PCR; (**C**) ENCODE contains data for 11 NRF1 Chip-Seq experiments conducted in GM12878, H1, Hela-S3, HepG2 (× 2), K562 (× 4), SK-N-SH, and MCF-7 cell lines. The table lists the identities of candidate cis-Regulatory Elements (cCREs) immediately adjacent to the transcription start site (TSS) of all four NatA genes and the number of positive hits for NRF1 binding among the 11 Chip-Seq experiments; (**D**) Example of Chip-Seq plots were strong binding of NRF1 is detected in the promoter of NAA10 but not of NAA20. Plots were generated using the WashU Epigenome Browser; (**E**) Heatmap displaying the coordinated response of NatA/E and proteasomal genes to treatment with the proteasomal inhibitor MG132 or vehicle control DMSO for 4 and 24 h (re-analysis of GSE141858 data)

One interesting association identified by our analysis was of the “Proteasome” pathway as being strongly enriched among the co-expressed transcripts for all four NatA/E subunits in both the CCLE and SEEK. The NatA complex is considered the most prominent eukaryotic NAT complex, Nt-Acetylating ~ 40% of proteins (Aksnes et al. 2019), while the proteasomal pathway is central in the turnover of cellular proteins. Examination of the SEEK database revealed the co-expression of the proteasomal and NatA/E transcripts in a large number of cancers including bladder (GSE3167), breast (GSE20271) and colorectal (GSE13067) (as an example see bladder cancer Fig. 4A).

We also noted the co-regulation of NatA/E subunits and proteasomal transcripts in non-cancer contexts, with the most common association observed in conditions of T cell activation (e.g. GSE32607, GSE39596, GSE36766, GSE14422 among others). We validated the NatA/E-proteasome association in activated T cells isolated from two human donors (Fig. 4B). Thus, the co-expression of NatA and proteasomal genes occurs also in the setting of T cell activation.

We next examined the Encyclopaedia of DNA Elements (ENCODE) Chip-Seq datasets to determine whether members of the nuclear erythroid 2-like family (NRF1-3) of transcription factors, which regulate mammalian proteasomal genes (Kamber Kaya and Radhakrishnan, 2021), could also be involved in NatA regulation. In ENCODE datasets NRF1, but not NRF2, binding was observed in candidate cis-Regulatory Elements (cCREs) immediately adjacent to the transcription start site (TSS) of all four NatA genes (Fig. 4C). Example Chip-Seq experiments from ENCOCE showing the strong binding of NRF1 to the promoter of NAA10 but not of NAA20 in MCF7 breast, HepG2 liver and K562 myelogenous leukaemia cell lines are shown in Fig. 4D. One of the established conditions were NRF1 drives the transcription of proteasomal genes is a compensatory response following proteasomal inhibition (Balasubramanian et al. 2012).Based on this we analysed a dataset from human breast cancer MCF-7 cells treated with the MG132 proteasomal inhibitor MG132 for 4 and 24 h (Fig. 4E). This analysis found the temporally coordinated induction of NatA/E and proteasomal genes, consistent with their common regulation by NRF1. Thus, NatA/E and proteasomal genes are co-regulated in both cancer and non-cancer cells, with NRF1 being one potential factor underlying this coordination.

### Connection Between the Rb/E2F axis and NAA40 Transcriptional Upregulation

**Fig.5:**
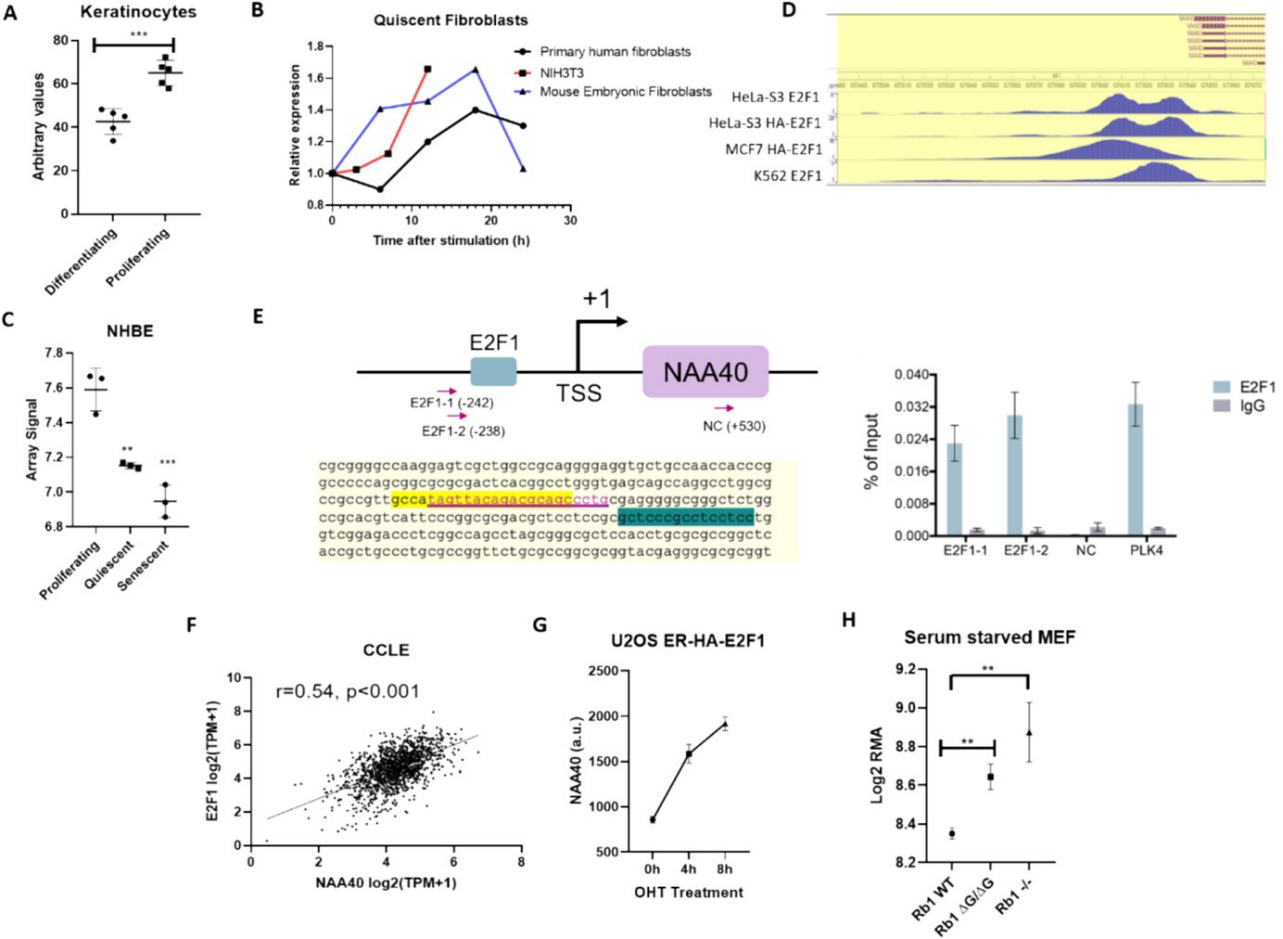
NAA40 transcript is enriched in proliferating cells. Comparison of NAA40 transcript levels in (**A**) Human keratinocytes isolated from five human volunteers and cultured under proliferating conditions (sub-confluent, low calcium media) or differentiating conditions (seven days of high calcium medium); (**B**) three independent studies of human and mouse fibroblasts following serum stimulation; (**C**) NHBE either in proliferating state or treated with the EGFR inhibitor Erlotinib to be forced into quiescence or senescence; (**D**) Chip-Seq data from ENCODE showing enrichment of E2F1 in NAA40 promoter (**E**) binding of E2F1 to the promoter of NAA40 in HCT116 cells. Left panel showing location of E2F1 primers. Right panel, amplification of genomic region following E2F Chip; (**F**) Scatterplot plotting *E2F1* (y-axis) and *NAA40* (x-axis) in the CCLE, each symbol represents a cell line, Spearman’s correlation is shown; (**G**) *NAA40* levels in serum-starved U2OS cells following E2F1 induction by 4-Hydroxytamoxifen treatment; (**H**) *NAA40* levels in serum-starved mouse embryonic fibroblasts (MEF) of Rb1 knockout (KO), Rb1 wildtype (WT) or in a Rb1 ΔG/ΔG genotype

Another interesting association revealed by our analysis was of NAA40 with the KEGG “DNA replication” and “Cell cycle” pathways. Currently the only known substrates of NAA40 are histones, the biosynthesis of which is associated with cell cycle commitment and the packaging of newly replicated DNA (Armstrong and Spencer, 2021). We considered it therefore plausible that the transcriptional induction of NAA40 occurs in highly proliferating cells. In order to investigate the potential connection between a more proliferative state and NAA40 levels, we identified datasets where defined treatments were used to manipulate the entry or exit of diverse cell types into the cell cycle, namely serum-starved fibroblasts, keratinocytes in high calcium media and Normal Human Bronchial Epithelial Cells (NHBE) treated with the EGFR inhibitor Erlotinib. Indeed, in all three examined conditions, the NAA40 transcript levels were higher in the proliferating compared to the non-proliferating cells (Fig. 5A–C). To identify potential factors that could be linking NAA40 transcription with DNA replication/cell cycle, we examined its promoter region (2000 bases upstream of the transcription start site) using the PROMO in silico tool. Interestingly, among the predicted transcription factor binding sites within the NAA40 promoter, we noted the presence of a canonical E2F1 motif in the genomic area immediately upstream to the NAA40 transcriptional start site. Examination of the ENCODE Chip-Seq datasets revealed E2F1 binding within this genomic area in Hela-S3, MCF7 and K562 cell lines (Fig. 5D), which we validated by Chip-qPCR in HCT-116 cells (Fig. 5E). Consistent with E2F1 being a transcriptional driver of NAA40, the two transcripts displayed highly significant positive correlation in the CCLE (Fig. 5F). Finally, we examined datasets from studies where E2F1 was manipulated in order to determine the subsequent effects on the NAA40 transcript. In the first study, (GSE61272) 4-Hydroxytamoxifen treatment- induced induction of E2F1 in serum-starved U2OS resulted in increased *NAA40* (Fig. 5G). In a second examined study (GSE54924), the retinoblastoma (Rb1) upstream repressor of *E2F1* was manipulated in serum-starved mouse embryonic fibroblasts. Analysis of the associated microarray data revealed that compared to WT MEF cells, *NAA40* levels were higher in cells lacking Rb1 or an *Rb1* ΔG/ΔG mutant which is unable to interact with E2F1 (Fig. 5H). Therefore, the Rb/E2F1 axis is likely involved in the transcriptional induction of NAA40 in normal proliferating and cancer cells.

### Protein-Level Regulation is more Prominent for Heteromeric NAT Complex Subunits Compared to the Monomeric NAA40

**Fig.6:**
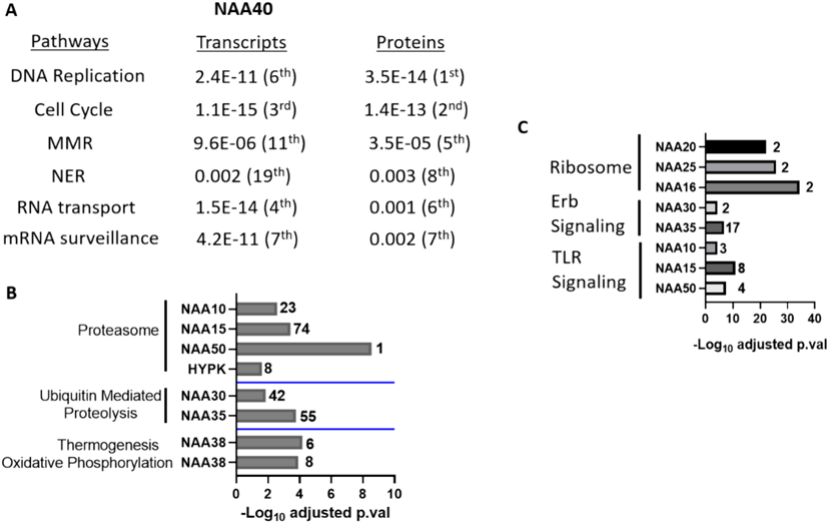
Selected examples of enriched pathways among NAT co-expressed proteins in the CCLE study. (**A**) Comparison of corrected p.values and relative ranks for pathways involved in DNA replication and repair among significantly co-expressed transcripts and proteins in the CCLE study for NAA40; Examples of pathways that are significantly enriched (**B**) among both co-expressed transcripts and proteins or (**C**) only among co-expressed proteins. The X-axis represents the –Log_10_ adjusted p.val, while the Y-axis represents the implicated NAT. The numbers next to the bar graphs denote the relative rank of the pathway among all KEGG pathways based on the significance levels of the enrichment

The increasing availability of proteomic datasets allows the investigation of regulatory processes that go beyond transcript level regulation (Jiang et al. 2020; Nusinow et al. 2020). To investigate the potential involvement of post-transcriptional processes on the regulation of human NAT, we repeated our previous analysis of pathway enrichment in the CCLE, but in this case utilising proteomic data generated for 375 cell lines (Nusinow et al. 2020). NAA60 was not detected in any samples and NAA11 in only 27 cell lines, so these NAT could not be investigated further. Notably, NAA40 stood out from among the examined NAT in displaying the highest similarity in the pathways enriched among either its significantly co-expressed transcripts or proteins. For both analysis, the list of the most significantly enriched pathways was dominated by those belonging to DNA replication, DNA repair and RNA processing (Fig. 6A). The greatest discrepancy involved the “Spliceosome”, which was the most significantly enriched pathway among co-expressed transcripts, but was not enriched among co-expressed proteins. Hence, for NAA40, our analysis revealed a high degree of concordance between transcript and protein co-expression profiles. A generally lower degree of concordance was observed for enriched pathways of co-expressed transcripts and proteins for the NatA-E complex subunits. In certain cases, NAT-pathway associations previously identified in the transcriptomic analysis were also valid in the analysis of proteomic datasets, although were relatively less prominent. Examples include NatA subunits with “Proteasome”, NAA30-NAA35 with “Ubiquitin Mediated Proteolysis” and NAA38 with “Oxidative Phosphorylation” and “Thermogenesis” (Fig. 6B). A number of strong NAT-pathway associations were also revealed specifically in the analysis of co-expressed proteins while not being observed in the transcriptomic datasets. Examples include that of NAA20-NAA25 with “Ribosome”, NAA30-NAA35 with “Erb signalling”, and NAA10-NAA15-NAA50 with “TLR signalling” (Fig. 6C). Finally, a number of associations were only significant in the transcriptomic but not in the proteomic analysis, such as of NAA15/NAA50/NAA25/NAA16 with “Cell cycle”.

This analysis therefore supports that post-transcriptional processes of regulation are more prominent for heteromeric NAT complexes. This motivated us to then examine the degree of concordance between transcript and proteins for each NAT in the CCLE. Consistent with the previous co-expression analysis, NAA40 had the strongest transcript-protein correlation (r = 0.5), while the correlation was also high for NAA80, NAA30 and NAA15 (r = 0.4). For other NAT, the transcript-protein correlations were low to none (r = 0–0.2 for NAA10, NAA20, NAA35, NAA38, NAA16), supporting the greater importance of post-transcriptional processes on their regulation.

### Coordination of the Abundance of NAT Complex Subunits Occurs Predominantly Through Post-Transcriptional Processes

**Fig.7:**
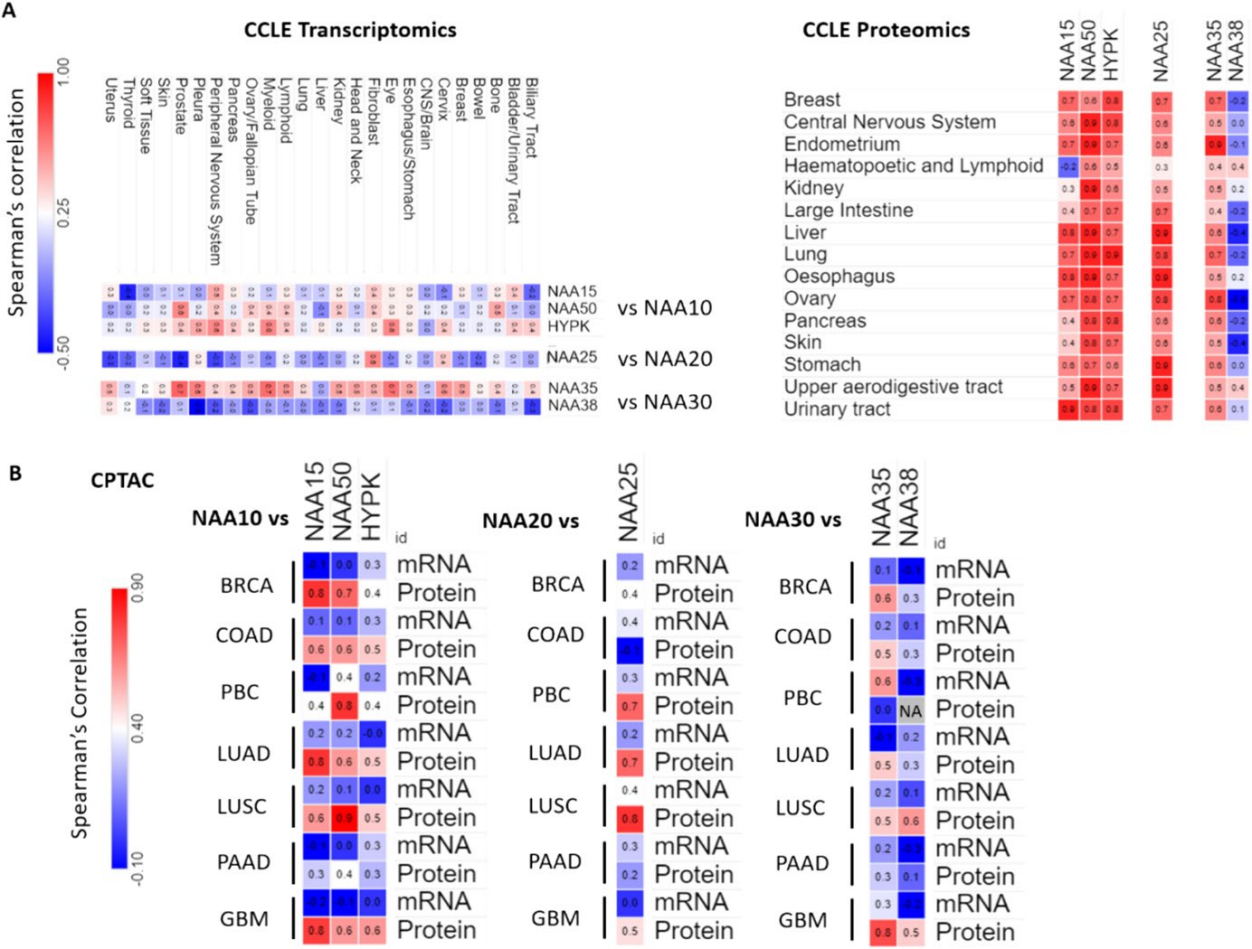
Comparison of correlations between components of the same NAT complex at the transcript and protein levels. Heatmaps displaying the Spearman’s correlations between catalytic and auxiliary subunits of NAT complexes at the transcript and protein levels in (**A**) CCLE and (**B**) CPTAC. In CCLE tissues represented by at least 10 cell lines are shown. Tumour abbreviations shown in figure: GBM glioblastoma, LUAD lung adenocarcinoma, BRCA breast cancer, COAD colorectal cancer, LUSC lung squamous carcinoma, PAAD prostate adenocarcinoma, PBC Paediatric brain

Maintenance of the appropriate stoichiometry of protein complexes in the presence of environmental or genetic perturbations occurs through either coordinated transcription or protein synthesis/degradation. Our previous comparison of transcripts and proteins from the CCLE indicated the prominence of post-transcriptional processes for controlling the levels of individual NAT complex subunits. Notably, calculating and comparing the correlation between the catalytic and accessory subunits of NatA, NatB and NatC in the CCLE revealed a prominently stronger association at the protein level compared to the mRNA level **(**Fig. 7A). For example, in the CCLE at the mRNA level, the range of Spearman correlation across cell lines originating from the same tissue for NAA20-NAA25 was from -0.4 to 0.6, with an average of 0, while at the protein level the range ranged 0.2–0.9 with an average of 0.7.

To validate this observation, we also examined data from the Clinical Proteomic Tumour Analysis Consortium (CPTAC), an initiative that has generated mass-spectrometry based proteomics and transcriptomic datasets levels across seven types of cancer: breast (BRCA); colon (COAD); lung adenocarcinoma (LUAD); lung squamous cell carcinoma (LUSC); Paediatric brain (PBC); pancreatic ductal adenocarcinoma (PAAD) and glioblastoma (GBM). Again, much stronger correlations were found between NAT complex subunits at the protein compared to the mRNA levels (Fig. 7B). It should also be noted that the correlations of complex components identified were generally among the highest calculated for the catalytic enzymes compared to all other proteins. For example, in BRCA, NAA15 and NAA50 were the top two most strongly correlated proteins with NAA10; NAA25 was the top most correlated with NAA20; and NAA35 the top most correlated with NAA30. As can be seen in Fig. 7A, the one exception to the generally high intra-complex correlation of NAT subunits at the protein level was NAA38. The reason for the apparent lack of coordination of NAA38 with the other subunits is currently not clear, since NAA38 is considered to be an obligate component of the NatC complex. Possible explanations could be that NAA38 had distinct functions and regulation compared to other two NatC subunits, that NAA30/NAA35 function also as a binary complex or that NAA38 levels are constitutively much higher than that of the other two subunits.Therefore, our analysis suggests that with the exception of NAA38 post-transcriptional regulation of intra-complex subunits enhances their coordination, potentially facilitating their assembly.

### Genomic Perturbation of NAT Multi-Component, but not of Monomeric, Complexes are Neutralised at the Protein Level

Somatic Copy Number Alterations (SCNA) are common events in cancers, whereby loss or amplifications of genomic regions affect the number of copies of genes contained within these regions. Interestingly, recently reported cancer studies which coupled transcriptomic and proteomic investigations of cancer tissues have revealed the capability of post-transcriptional mechanisms to neutralise the effects of SCNA on the protein level, despite the gene dosage effect being observable at the transcript level (Krug et al. 2020). Given our previous analysis, we considered it plausible that similar protein level control of NAT abundance would be active in cancers where SCNA affect NAT complex components. To examine this hypothesis, we identified CPTAC cancers with appreciable detection of SCNA events affecting NAT complex subunits, and examined the consequent effect on mRNA and protein levels. For ease of interpretation of the impact of SCNA on examined NAT, we have plotted the Z-score values for mRNA and proteins, although the results are equivalent when plotting absolute values.

**Fig.8:**
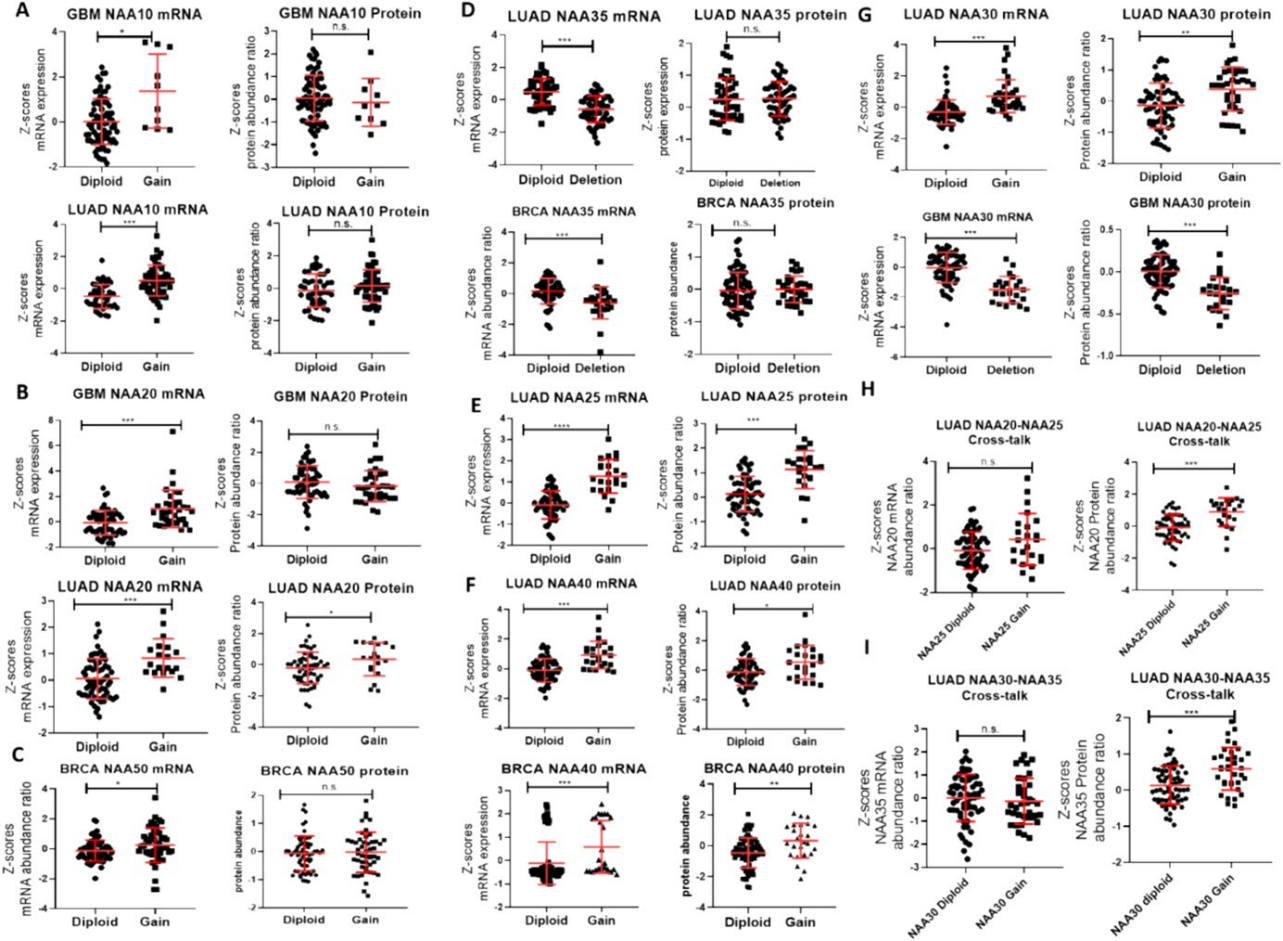
Effect of copy number alterations on NAT transcript and protein levels. (**A-G**) The CPTAC collection of tumours with coupled transcriptomic and proteomic datasets was examined to identify cancer samples with significant gain or losses of NAT genes. For each cancer with identified somatic copy number alteration of a NAT gene, a pair of plots are depicted, one representing the effect on the transcript level (Y-axis = Z-score mRNA expression) and one representing the effect on the protein level (Y-axis = Z-scores protein abundance). The X-axis groups samples into Diploid and either Gain (increased gene copy numbers) or Deletion (decreased copy number). Each symbol in the plots represents one cancer sample, and in orange colour, the median and standard deviations are shown. * *p* < 0.05, *** *p* < 0.001 Mann Whitney test. Tumour abbreviations shown in figure: GBM glioblastoma, LUAD lung adenocarcinoma, BRCA breast cancer; (**H,I**) The impact of NAA25 and NAA30 copy number alterations on the mRNA and protein of NAA20 and NAA35, respectively, are shown

For NAA10 and NAA20, gains in copy numbers in GBM and LUAD led to significant increases of their transcripts, but not of their proteins (Fig. 8A, B). A similar pattern was observed for NAA50 gene gain in BRCA (Fig. 8C) and for deletions of the NAA35 gene in BRCA and LUAD (Fig. 8D). Thus, for these NAT, our analysis supports the involvement of protein-level regulation in neutralising genomic alterations. Conversely, we identified instances with concordant increase in both transcript and protein levels for NAA40, NAA30 and NAA25 (Fig. 8E–G). The high degree of concordance for NAA40 in the effect of SCNA on both the transcript and protein levels indicates that post-transcriptional processes are not predominant for this monomeric NAT. However, a more complicated picture emerged for NAA30 and NAA25, as we noted that we had noted previously that their complex partners NAA35 and NAA25, respectively, displayed evidence of post-transcriptional buffering of gene dosage effects. We therefore considered the possibility of homeostatic post-transcriptional regulation for NAT complexes, with NAA20 and NAA35 “sensing” and responding to the levels of the NAA25 and NAA30, respectively. Indeed, we observed that in GBM, tumours with gain in the copy numbers of NAA25, the protein levels of NAA20, but not its transcript levels, were significantly increased (Fig. 8H). A similar observation also occurred in LUAD where copy number gains of NAA30 resulted in increased levels of NAA35 specifically at the protein level (Fig. 8I). In conclusion, examination of SCNA events in human cancers offers further evidence for the importance of post-transcriptional regulation on controlling levels for some NAT, in a manner that facilitates the required stoichiometry of its components. For the monomeric NAA40, such mechanisms do not appear to be active for homeostatic control of its gene dosage.

## Discussion

Expression of protein-coding genes can vary considerably from ubiquitous to highly tissue-specific, depending on their biological functions. Genes that are found in all tissues and cells, and at comparable levels, tend to perform tissue-independent basic cell activities, such as ones relating to cell maintenance (Eisenberg and Levanon 2013; Jin et al. 2023). Conversely, a large number of genes are characterised by tissue-enriched/specific expressions, and transcriptomic profiles can distinguish individual tissues (GTEx Consortium 2015; Jin et al. 2023). In CRISPR screens, NAT genes were in the top 80–99% of the most essential genes across cells, with the exception of NAA60, NAA80 and the paralogs NAA11/NAA16 (Koufaris and Kirmizis 2020). This observation supports general tissue-independent functions for NATs. On the other hand, hereditary mutations affecting the NatA and NatB complexes are associated with phenotypes in specific tissues/organs (McTiernan et al. 2022; Morrison et al. 2021), which could indicate tissue-specific functions. Moreover, different amounts of NAT complexes could theoretically be required in a given tissue depending on the relative level of their substrate targets. Nevertheless, cross-tissue comparison of NAT transcript and protein levels performed here revealed a general consistency for these complexes (Fig. 2). Therefore, it appears that tissue-specific phenotypes relating to NAT activity are probably due to their effects on specific substrates within those tissues, irrespective of the abundance of these complexes. NAA11 was the only tissue-specific human NAT, being detected only in the testis and the placenta. The presence of NAA11 in the testis has been proposed to act to compensate for reduced levels of its X-linked paralog NAA10 in this organ (Pang et al. 2011). We also note here for the first time the detection of this transcript in a tissue beyond the testis, namely in the placenta, suggesting it is also needed in this tissue. In contrast to NAA11 and NAA10, the second set of human NAT paralogs (the auxiliary NAA15/NAA16) was both consistently detected across all tissues, raising the possibility that these perform non-redundant functions relating to NatA activity. Finally, the NAA80 transcript was revealed to be testis-enriched in all three of the examined human transcriptomic datasets. Although unclear at present, the need for highly enriched NAA80 in the testis could relate to distinct functions of its target actin in this organ, for example, its role in the formation of the blood-testis barrier (Cheng and Mruk, 2012) or alternatively in the targeting of currently unidentified testis-enriched/specific protein.

Since human NAT complexes differ in their specificities, substrate repertoires and cellular localisation, a reasonable expectation is that they will be subjected to distinct regulation, in order to more efficiently execute their differential functions. Our analysis in this study revealed clear differences in the association of cellular pathways with human NAT, with notable examples the association of NatA with the proteasome and of NAA40 with DNA replication. The proteasome is a multi-subunit complex catalysing the degradation of damaged, misfolded and unwanted proteins. Because a large number of proteins involved in many biological processes are subjected to homeostatic proteasomal degradation, the activity of these pathways is tightly regulated. Despite Nt-Ac being clearly linked to protein turnover, it has paradoxically been reported to both increase and decrease protein stability (Kats et al. 2022; Shemorry et al. 2013; Varland et al. 2023). The co-regulation of the NatA and the proteasome complexes occurs in both cancer and non-cancer cells, with NRF1 potentially being the transcription factor underlying this connection. An interesting hypothesis is that the organismal benefit to the co-regulation of these two complexes could be to protect specific subsets of proteins from tagging and degradation in conditions of increased proteasome activity, although this needs to be tested in future studies. For NAA40, we noted its association with DNA replication, a highly regulated process whereby a dividing cell replicates its entire genome. In eukaryotic cells, DNA is wrapped around histones to form nucleosomes, the basic unit of chromatin. Consequently, the interaction between histones and DNA is a central aspect of the process by which the genome can be replicated and repackaged. Currently the only known substrates of NAA40 are two of the four core nucleosome histones, H2A and H4, with the Nt-Ac of these proteins considered to be both highly abundant and irreversible (Demetriadou et al. 2020). Considerable amounts of the core histones are required to restore duplicated chromatin during the “S” phase of the cell cycle, with the enriched production achieved through increased transcription and half-life of their mRNAs (DeLisle et al. 1983; Heintz et al. 1983). One possible explanation therefore for the association of NAA40 with DNA replication is that transcriptional induction of this NAT is required to achieve sufficient Nt-Ac of H2A and H4. Importantly, we demonstrated here the binding of E2F1, a main transcription factor activated by growth factors to drive entry into the cell cycle (Ertosun et al. 2016), in the promoter of NAA40. Over-activation of E2F pathway is also known to be common in human cancers (Chen et al. 2009), and could potentially underlie the upregulation of the NAA40 transcript in several tumour types (Koufaris and Kirmizis 2020).

Another important insight of this study is the prominent role of post-transcriptional control for coordinating the abundance of NAT complex subunits. Achieving and maintaining the correct stoichiometry of multi-subunit protein complexes are essential for maintaining their functional integrity and structural stability. This is achieved through co-regulated transcription and/or post-transcriptionally through control of translation and/or degradation rates of protein complex subunits (Shemorry et al. 2013; Taggart et al., 2020). Here, we have not investigated further how this post-transcriptional coordination occurs, which likely involves altered rates of mRNA translation or protein degradation. Irrespective of this, caution is required when investigating the role of NAT in physiological and disease states to also examine protein abundances, especially for the multi-subunit complexes.

Since this study was designed as an initial survey into the regulation of the multiple human NAT complexes, it necessarily has limitations. At the same time, the insights obtained from this study suggest interesting avenues for further investigations. A first limitation of this study was that NAA60 was not detected in proteomic datasets, possibly due to its localisation within the membrane of the Golgi. Investigations of protein-level regulation of NAA60 would require the measurement of the protein levels of this NAT through alternative approaches. A second limitation is that while our comparison of the intra-complex subunit correlations at the transcript and protein levels revealed the predominance of post-transcriptional processes, this approach does not offer insights into mechanisms through which this coordination occurs. Such mechanisms insights could be achieved through experimental investigations, for example, transient repression of NAT subunits coupled with measurement of the impact on mRNA translation and protein degradation rates. Finally, while co-expression and co-regulations can potentially indicate important biological functions for NAT, these need to be experimentally investigated. Such interesting cases of potential novel biological functions of human NAT complexes identified here include the induction of NatA/E in activated T cells and of NAA40 with DNA replication.

## Conclusion

Of note, Archaea contain a single NAT ortholog that is sufficient to fulfil the protein Nt-Ac requirement in these organisms (Liszczak and Marmorstein 2013). The eukaryotic lineage has therefore seen the prominent expansion of the NAT family, allowing the specialisation of the novel complexes towards divergent substrates and/or cellular locations. As we show in this study, intra- and inter-complex transcriptional and post-transcriptional regulation is crucial in achieving the desired versatility and optimal functioning of these new eukaryotic NAT complexes.

## Methods

### Public Data Acquisition and Processing

To examine and compare NAT transcript levels across normal human tissue types, we utilised publicly available transcriptomic datasets collected as part of three consortiums: GTEx, HPA and FANTOM. For GTEx Normalised TPM, data (V8) for GTEx samples were downloaded from the GTEx portal (https://www.gtexportal.org/home/datasets). Transcriptomic data for HPA (normalized expression ("nTPM") rna_tissue_hpa.tsv.zip) and FANTOM (normalized expression (“nTPM”) rna_tissue_fantom.tsv.zip) were obtained from HPA portal (https://www.proteinatlas.org/about/download). Normalised proteomic datasets for 375 cell lines were previously generated by the Gigy lab and were obtained from the Depmap portal (https://depmap.org/portal/download/all/). Proteomic data for normal human tissues were obtained from the supplementary data of Jiang et al. 2020 paper. For each study, the median NAT transcript or protein level was determined in each tissue, followed by calculation of Z-scores. Tissue outliers for either transcripts or proteins were defined as those with Z-scores ± 3 across all tissues.

For co-expression analysis, the CCLE study transcripts and proteins were ranked for each NAT according to their Spearman’s correlation value across all samples from highest to lowest. For the proteomic dataset, proteins which were detected in less than 20% of the examined cell lines were excluded from further analysis. Significantly correlated transcripts and proteins (r > 0.3 and Benjamini Hochberg corrected p.val < 0.05) were then passed onto Enrichr (https://maayanlab.cloud/Enrichr/) for enrichment analysis within Kyoto Encyclopaedia of Genes and Genomes collection of datasets, using KEGG genesets with a minimal of 15 genes and maximum of 500. Significantly enriched pathways were considered those with adjusted p.values < 0.05. For SEEK, default settings were first used to rank genes according to their co-expression with NAT genes, weighed across a compendium of more than 3000 transcriptomic datasets. The top 1000 ranked genes for each NAT were then used for enriched as described previously.

Processed CPTAC data were downloaded from cbiolportal (https://www.cbioportal.org/datasets). For protein–protein co-expressions, Spearman’s correlations were calculated for each NAT gene against proteins and p.values adjusted for multiple testing by using Benjamini Hochberg correction. Significantly positively correlated proteins (r > 0.3, adj,p.val < 0.05) or negatively correlated (r < -0.3, adj,p.val < 0.05) were used for KEGG enrichment analysis using EnrichR. For Somatic Copy Number Alterations (SCNA), the copy number for NAT genes was extracted from pre-processed GISTIC algorithm generated estimations for the CPTAC studies. GISTIC values of “1” were considered Gains, “0” as diploid and “-2” as Deletion.

Transcriptomic datasets were obtained directly from the NCBI GEO archive. Where multiple probes were present the average value was taken. No further normalisation was performed.

### T Cell Isolation and Activation

Human T cells were isolated from peripheral blood samples taken from healthy consented volunteers following procedures approved by the Cyprus National Bioethics Committee. Firstly, peripheral blood mononuclear cells were isolated using density gradient centrifugation. Briefly, the blood sample was diluted 1:1 with sterile PBS and carefully layered on top of Lymphosep medium. The sample was then centrifuged at 400 g for 30 min at 4 °C. The layer containing the mononuclear cells was carefully aspirated and used for the isolation of T cells. Human T cells were isolated using the EasySep™ Human T Cell Isolation Kit (Catalog #17,951, STEMCELL Technologies), based on manufacturer's instructions. Briefly, mononuclear cells were prepared at 5 × 10^7^ cells/mL (0.25-2 mL), mixed with 50 μl/mL of Isolation Cocktail and incubated for 5 min at RT. Subsequently, 40 μl/mL of RapidSpheres were added, gently mixed and volume topped up to 2.5 mL. The tube containing the mixture was placed on a magnet and left for 3 min at RT, at which time purified cells were carefully transferred into a new tube. Total T cell count was calculated using a hemocytometer. For T cell activation, cells were resuspended in ImmunoCult-XF T Cell Expansion Medium (Catalog #10,981, STEMCELL Technologies) supplemented with 2 mM L-Glutamine, 50 μg/mL penicillin/streptomycin, and 10 ng/mL Human Recombinant IL-2. Cell density was adjusted to 1 × 10^6^ cells/mL and 25μL/mL of ImmunoCult Human CD3/CD28 T Cell Activator (Catalog #10,971, STEMCELL Technologies) was added. T cell activation was confirmed through assessment of cell proliferation and expression of activation markers. After incubation for the indicated amount of time, RNA was isolated for further analysis.

### qRT-PCR

Total RNA was extracted using the RNeasy Mini kit (Qiagen) according to the manufacturer’s instructions. Total RNA was then reverse transcribed to complementary DNA using the PrimeScript RT reagent kit (Takara) with random primers. qRT-PCR was carried out using KAPA SYBR Green (SYBR Green Fast qPCR Master Mix) and the Biorad CFX96 Real-Time System. Expression data were normalized to the mRNA levels of the β-actin housekeeping gene and calculated using the 2 − ΔΔCt method. Primers used were ΝΑΑ10 F-TGCTGAGGACGAGAATGGGAAG, NAA10 R-CTGGTCCATCAGTTTCTGAGCC; NAA50 F-GAGGTTGGCGAGCTAGCAAAAC, NAA50 R-TAGCCTTCGGTAAGGTGCCAGA; PSMA1 F- AACAAGGTTCAGCCACAGTTG, PSMA1 R- ACACAGGCAGTGGTCTATCG; PSMD13 F- AGCCTCTCATCCGTTTTTCACT, PSMD13 R- AGAGCCACATTAGGATCAGTCAT; ABL F-AAGCCGCTCGTTGGAACTC, ABL R-AGACCCGGAGCTTTTCACCT.

### E2F Chip

To perform Chip for E2F, we followed the protocol by Lee et al. (Lee et al. 2018). Briefly, HCT116 cells were first fixed in 1% formaldehyde and quenched with 125 mM glycine. Next, the cells were lysed in SDS lysis buffer (1% SDS, 10 mM EDTA, 50 mM Tris–HCL pH 8 and protease inhibitor cocktail) followed by DNA sheared using a Bioruptor sonicator (Diagenode). The sheered chromatin was then diluted tenfold in IP buffer (1% Triton-X-100, 2 mM EDTA, 50 mM Tris–HCL pH 8, 150 mM NaCl and protease inhibitor cocktail) followed by 1 h preclearing using Protein A sepharose beads (GE Healthcare) at RT and incubation with 1 μg of antibodies against E2F1 (Cat. No. 3742 Cell Signalling) or IgG (Biogenesis 5180–2104) for 1 h at 4 °C. Next, 50% slurry protein A beads blocked in salmon sperm DNA were added and incubated overnight at 4 °C. Following washing steps, the immunoprecipitated chromatin was eluted in freshly prepared elution buffer (1% SDS and 0.1 M NaHCO3) and reverse cross-linked using 200 mM NaCl containing 0.5 μg/μl RNase (Roche) at 65 °C overnight. The samples were purified using the QIAquick PCR purification kit (QIAGEN) and analysed with qRT-PCR using two primer sequences for E2F1, PLK4 as positive control, and a negative control region within NAA40 ORF. E2F1 First set: For-CTCTGGCCGCACGTCATT and Rev-CATGCGCCTCGCAGCTT; E2F1 Second set: For-CGGCGCGCGACTCAC and Rev-GGCTGCGTCTGTAACTATGGC; Negative Control region For- TGACTTTGGAGCCCGAGGTA and Rev- GCCAACTCACTGGCACACTA; Plk4 For-AGT GTCCCGAGGCACTGCGGCTT, Plk4 Rev -AGATAACCGCCATCCCCTTGGA.

## Abbreviations

BRCA: Breast cancer
CCLE: Cancer Cell Line Encyclopaedia
cCREs: Candidate cis-Regulatory Elements
ChiP: Chromatin Immunoprecipitation
COAD: Colorectal cancer
CPTAC: Clinical Proteomic Tumour Analysis Consortium
ENCODE: Encyclopaedia of DNA Elements
GTEx: Genotype-Tissue Expression
HPA: Human Protein Atlas
FANTOM: Functional ANnoTation Of the Mammalian genome
GBM: Glioblastoma
KEGG: Kyoto Encyclopaedia of Genes and Genomes
Nt-Ac: N-terminal acetylation
LUAD: Lung adenocarcinoma
LUSC: Lung squamous cell carcinoma
NAT: N-terminal acetyltransferases
SEEK: Search-Based Exploration of Expression Compendium
TPM: Transcripts per million
PBC: Paediatric brain
PAAD: Pancreatic ductal adenocarcinoma
